# A Brief (If Insular) History of the Human Genome Project

**DOI:** 10.1371/journal.pbio.1000601

**Published:** 2011-03-08

**Authors:** Michael J. Morgan

## Abstract

Michael Morgan reviews *Drawing the Map of Life*, an American journalist's view of the international sequencing effort.

**Figure:**
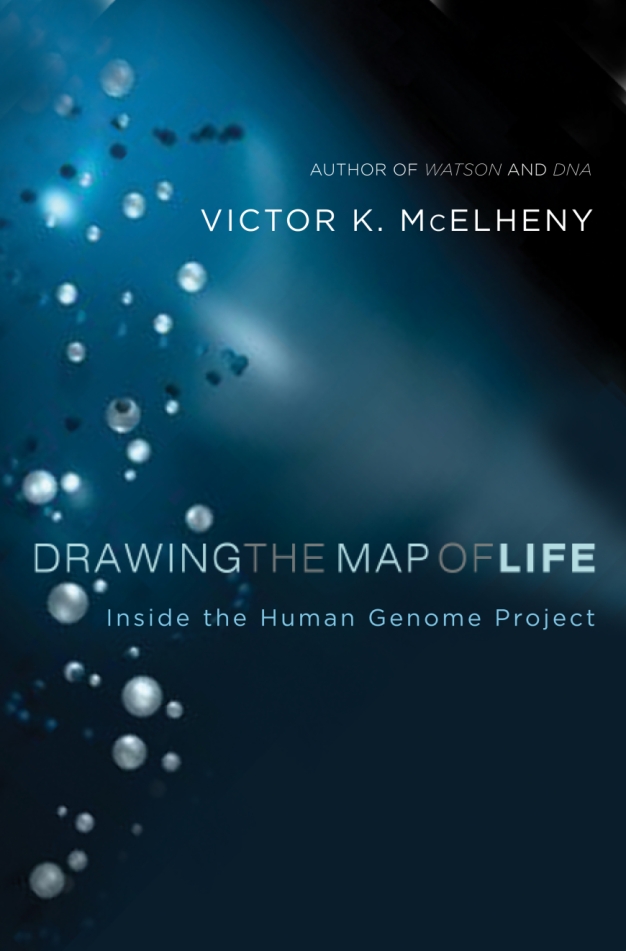
McElheny VK (2010) Drawing the Map of Life. New York: Basic Books. 384 p. ISBN 978-0465043330 (hardcover). US$28.00.

September 2010 was the 70th anniversary of the Battle of Britain. A number of commentators, historians, and other pundits have debated the significance of the battle: was it a truly significant turning point (or tipping point in present parlance) in World War II? Similarly, historians of science and others will no doubt be mulling over the true significance of the human genome sequencing project 70 years hence and thereafter. In *Drawing the Map of Life*, veteran science journalist and author Victor McElheny offers a view “inside the human genome project” that covers the origin of the project in the United States, its development and implementation, and its impact since its “completion” in 2000. It is a hugely readable account that gives the reader a sense of the excitement and drama that gripped the main protagonists along with a description of the technological advances that made the project feasible and affordable. It relies heavily on interviews with a number of scientists, mainly from the US, newspaper articles, and radio and television reports, but pays scant attention to the international character of the project. It is important to reaffirm that one country did not do it alone; it was an international effort and the many partners are justifiably proud of their contribution.

There seems little controversy that it was Robert Sinsheimer, then chancellor of the University of California at Santa Cruz, who was the first to call a meeting (in May 1985) to discuss the feasibility of sequencing the human genome. As related by McElheny, among the dozen or so scientists at the meeting was John Sulston from the Laboratory of Molecular Biology (LMB) in Cambridge, United Kingdom, who published his own account of the project in 2002, a year before the completion of the sequence was declared [1]. John, with Bob Waterston of Washington University, St. Louis, were arguably the first to invest heavily in the sequencing of an animal genome, *Caenorhabditis elegans*, funded jointly by the Medical Research Council (MRC) in the UK and the National Institutes of Health in the US. This was a unique partnership championed by the Secretary of the MRC, Sir Dai Rees, and Jim Watson, Director of the National Center for Human Genome Research at the National Institutes of Health, respectively. When John and Bob were being solicited by Frederick Bourke to join his private effort to sequence the human genome, it was not surprising that Watson alerted the MRC to the danger of losing their star sequencer and an approach was made to the Wellcome Trust in the UK to help fund a human genome sequencing programme.

The Trust responded in 1992 by not only agreeing to join the MRC in supporting an initiative that would play a role in mapping, sequencing, and decoding the human genome and the genomes of other organisms, but also, for the first time in its history, to establish its own research station, now called the Wellcome Trust Sanger Institute at Hinxton, Cambridgeshire. This decision was not due to a change of strategy by the Wellcome Trust, but was a pragmatic response to the need to get a large sequencing facility up and running as soon as possible. John Sulston and I visited a number of possible sites, including a poultry research centre where John thought the chicken sheds would provide adequate housing for sequencing! A number of universities were considered, but it became clear that none were likely to be able to provide suitable accommodation in a timely fashion. John identified a site at Hinxton, Cambridgeshire, formerly a Tube Investments plc engineering research station, purchased by Capital and Counties plc, who had ambitious plans for a business park—a venture that never got off the ground. (At the time, there were ill-founded rumours that Tube Investments had been involved in the UK’s nuclear weapons research.) The Trust acquired the site in the autumn of 1992 and the metallurgy laboratories were re-fitted as a state-of-the-art sequencing facility in a period of a few months. The first new occupants moved in during March 1993, and by the end of the year there were over 80 staff on site and space was tight. In October 1993, the facility was formally opened and named by Fred Sanger as the Sanger Centre.

In a chapter called “Building the Toolbox”, McElheny describes the recombinant DNA technologies developed by Hamilton O. Smith, Fred Sanger, Walter Gilbert, Kary Mullis, and Lee Hood, amongst others, that enabled the manipulation of DNA, its amplification and sequencing, and the development of automated sequencers. He then goes on to describe some of the early efforts to garner public support (and funding) of the project, particularly in the US, and the role of Jim Watson in convincing Congress to fire the starting pistol.

John, Bob, and many of their colleagues were determined that the effort should be international in scope and involve all laboratories able to engage in large-scale sequencing; they were also concerned at the apparent lack of cooperation in the human genetics community (in contrast to their experience with the worm community). To promote international coordination and cooperation, the Wellcome Trust decided to host a meeting to which all relevant parties would be invited. It was decided to hold the meeting on “neutral ground” in Bermuda, and in February 1996, the first meeting of representatives from sequencing centres and funding agencies around the world met and established a coordinated effort, in the public domain, to sequence the human genome. Some 50 or so scientists and administrators from the US, UK, France, Germany, and Japan (joined a year or so later by China) discussed the scientific strategy for a distributed sequencing effort and, in particular, agreed on a policy for the release of data generated by the project, the so-called Bermuda Principles [2]. The principles evinced the benefits of the immediate release of raw data on the Internet without any privileged “first sight or use” and to pledge not to seek patent protection. These principles did not receive universal acclaim, and caused consternation among a few groups who were concerned that their institutions, funding bodies, or governments might be unwilling to agree to the data release guidelines. However, it was made clear that continued membership in the human genome sequence “club” required agreement to the principles. In the end, these misgivings proved unfounded. These data release principles helped assuage the impression that a small cabal of privileged and massively funded researchers would be given an unprecedented advantage over the rest of the scientific community. The principles have been extended to many other large-scale collaborative projects in biology, such as the SNPs Consortium (TSC) and the Structural Genomics Consortium, and have driven scientific progress and industrial application alike.

McElheny covers the “race” between the public consortium (managed by Francis Collins of the National Human Genome Research Institute, Ari Patrinos of the Department of Energy, and myself of the Wellcome Trust) and the private company, Celera, headed by Craig Venter. It is well documented how Craig broke ranks and decided to commercialise the sequence and seek patent protection on a number of genes. As a result, the “race” to sequence the human genome began. It is perhaps salutary to report that eventually Celera deposited its sequence in GenBank and the human sequence and that of many other organisms are freely available to anyone with access to the Internet.

McElheny's account admirably covers the period from the early days of recombinant DNA (1960s) through to 2010 and the days of the exploitation of the knowledge garnered from the project. There is significant debate in the media, no doubt reflecting that 2010 is the 10th anniversary of the release of the “working draft” of the genome about the worth of the project. One camp, notably espoused by Craig Venter in a recent article in *Der Spiegel* [3], casts doubts on the (medical) worth of the project. McElheny seems to be on the other side, admitting that many benefits lie in the future, but that some, for example diagnostic tools in treatment of some cancers and in the emerging field of pharmacogenomics, are already part of good medical practise in America. It is comprehensive, but, inevitably, superficial in its treatment of many of the social and political aspects of the project. For example, the early role of the Human Genome Organisation (HUGO), the grassroots organisation established by the early pioneers in genomics, does not even get a mention. The inevitable stresses and strains that beset the public programme and the behind the scenes negotiations that led to the “Clinton-Blair” joint statement in 2000, in which they applauded the decision to rapidly release human DNA data into the public domain, are yet to be related. This is not, therefore, a definitive history of the human genome project, or even a definitive history of the American contribution to the project. It is, however, a very entertaining and well-written account of the material it covers and provides, unusually for a “popular” treatise, a comprehensive series of notes to sources (some 24% of the book!) that will lead the keen student into deeper studies.

**About the Author**

Dr. Michael Morgan is a consultant for scientific affairs and is involved in the Human Genome Archive Project at Cold Spring Harbor. Recently, he was Chief Scientific Officer at Genome Canada. As chief executive of The Wellcome Trust Genome Campus, Hinxton, Cambridge, he played a major role in the international coordination of the Human Genome Project. As director of Ventures and Partnerships at the Wellcome Trust, he was instrumental in the SNPs Consortium (TSC), a partnership with 12 industrial partners; DIAMOND, a new third generation synchrotron in Oxfordshire, a joint project with the UK Office of Science and Technology; and the Structural Genomics Consortium, a partnership with Canadian and Swedish public entities and pharmaceutical companies that is tasked with determining the structures of human proteins of importance to human health. All of these partnerships are dedicated to putting their data into the public domain as early as possible and with no restrictions on use. He is presently working for INSTRUCT (a European Infrastructure Project) coordinated by Oxford University.

## References

[1] Sulston J, Ferry G (2002). The common thread: a story of science, politics, ethics, and the human genome..

[2] The Wellcome Trust (1997). Statement on genome data release.. http://www.wellcome.ac.uk/About-us/Policy/Policy-and-position-statements/WTD002751.htm.

[3] von Bredow R, Grolle J (29 July 2010). SPIEGEL interview with Craig Venter. ‘We have learned nothing from the genome.’. Der Speigel.

